# Paternal thrombophilia and recurrent implantation failure: an exploratory case-control study

**DOI:** 10.5935/1518-0557.20240026

**Published:** 2024

**Authors:** Sedighe Esmaeilzadeh, Omid Jazayeri, Mir Mohammad Reza Aghajani, Shima Soleimani Amiri, Masoumeh GolsorkhtabarAmiri, Maryam Abdolahzade Delavar, Parvaneh Mirabi

**Affiliations:** 1 Infertility and reproductive health research center. Health Research Institute. Babol University of Medical Sciences, Babol, Iran; 2 Department of Molecular and Cell Biology, Faculty of Science, University of Mazandaran, Babolsar, Iran; 3 Razi Pathobiology and Genetic Diagnostic Laboratory, Babol, Iran

**Keywords:** recurrent implantation failure, thrombophilia, factor V Leiden, antithrombin III, protein C, protein S

## Abstract

**Objective:**

Many pieces of literature have reported that inherited and acquired thrombophilia might be a risk factor for recurrent implantation failure (RIF), however, most studies have only focused on RIF patients and not their male partners. We studied the possible association of paternal thrombophilia with RIF risk.

**Methods:**

Forty-two male partners aged 20-45 suffered from RIF compared with 42 males from couples with at least one successful pregnancy. All participants were investigated for thrombophilia markers.

**Results:**

The prevalence of coagulation Factor V activity was significantly higher in the case group (42.9%) than in the control group (16.7%) (*p*=0.008) (OR=3.75; 95% CI, 1.38, 10.12). The prevalence of protein C and protein S deficiencies in RIF patients were 4.8% and 2.4%, respectively, and 0% in the controls. The prevalence of antithrombin III (ATIII) deficiency was significantly higher in the case group (19%) than in the control group (2.4%) (*p*=0.01). None of MTHFR C677T and MTHFR A1298C were statistically significant between the two groups. Combined thrombophilia was 45.2% in the men of the RIF group when compared with the control, 14.2% (*p*=0.001) (OR = 4.95; 95% CI, 1.75-13.86).

**Conclusions:**

Paternal thrombophilia may be related to recurrent implantation failure, so evaluation of this factor in RIF patients could be used to identify relevant risk groups and may help in the proper management of these cases to enhance the chance of implantation.

## INTRODUCTION

Pregnancy rates reduce from 88% to 69% after two cycles, and from 87% to 53% after three cycles of IVF (Somigliana et al., 2018). Even in the case of high-quality embryo transfer, implantation is not definitive. Repeated Implantation Failure (RIF) is defined as failure implantation in > three consecutive high-quality embryo transfers or transfer of >10 embryos in multiple transfers and it includes <5% of couples undergoing IVF/ Intra cytoplasmic sperm injection (ICSI) (Bashiri *et al*., 2018). Apart from many risk factors involved in RIF, the activity of microvascular thrombosis and localized vascular impairment during ovarian stimulation for IVF in infertile women is associated with thrombotic events and suggested as a potential reason for RIF (Younis *et al*., 2014).

In a systematic review, women experiencing ≥1 assisted reproductive technique (ART) failure showed a greater risk of at least one inherited thrombophilic factor than those who had a live birth after IVF/ ICSI (Di Nisio *et al*., 2011). As the interval from embryo transfer to arterial thromboembolism is 3-28 days necessity to use thromboprophylaxis for women suffering RIF is still being debated (Tanacan & Beksac, 2019).

Given that both maternal and paternal genes contribute to the fetus and fetal membranes, the effect of thromboembolic mutations inherited from the father is worth considering. The contribution of paternal genes to the fetus may be critical within the pathophysiology of RIF, and paternal genes may have a crucial role in implantation. Incomplete invasion of the trophoblast or other abnormalities of placentation may be paternally derived characteristics (Manna *et al*., 2022). In a study on male factor V Leiden carriers, the interval between marriage and birth of a first child was significantly increased compared with non-carrier males. (van Dunné *et al*., 2006). Likewise, in 157 couples with unexplained infertility selected from five IVF centers, the incidence of carrier status for ANXA5 haplotype M2 was 44% of couples (one or both partners) (24% of women, and 26% of men) (Fishel *et al*., 2014). However, another study did not show any significant differences in factor V Leiden (FVL: G16916A) and prothrombin (factor II: G20210A) genotypes between infertile azoospermia or oligozoospermia men and normal controls (Yapijakis *et al*., 2016).

Little research to date, has tended to focus on male thrombotic carriers in recurrent pregnancy loss (Toth *et al*., 2008; Udry *et al*., 2014). To the best of our knowledge, this study is the first to elucidate the association of paternal thrombophilia with RIF risk.

## MATERIAL AND METHODS

This case-control study was performed in the Fatemeh Zahra Specialized Infertility Center. The protocol of the study was approved by Institutional Review Board at Babol University of Medical Sciences (No. IR.MUBABOL.REC.1400.151).

42 Iranian male partners of couples who suffered from RIF, aged 20-45 years old, were recruited as a case group. RIF was defined as the failure of implantation after three or more consecutive IVF/ICSI-ET cycles with all good-quality embryos. All fertilizations were carried out by ICSI.

*All couples* with *RIF* were recruited from Fateme Zahra Infertility and Reproductive Health Center affiliated to Babol University of Medical Sciences. After the approval of the ethics committee and before enrollment in the study, informed consent was obtained from all eligible participants by a gynecologist.

All subjects were screened for immunologic risk factors (anti-nuclear antibodies, lupus anticoagulant/antiphospholipid antibody) and excluded from the study if they had positive results. To rule out anatomical abnormalities, several imaging modalities were used. Sonographic, hysteroscopic, and laparoscopic reports, clinical examination, and laboratory results were assessed. Also, hormonal evaluation (T3, T4, TSH, FSH, LH, PRL) and thrombophilia markers were tested in the female partners to exclude any potential maternal causes of RIF.

Among the initial 65 subjects who were recruited for the study, 23 patients who had anatomical or chromosomal abnormalities, hormonal causes for RIF, or antiphospholipid syndrome were excluded from the case group, resulting in 42 RIF couples for the current study.

A control group of 42 males from age-matched couples with normal karyotype (46, XY), and with at least one successful pregnancy, without any complications (such as miscarriage, preeclampsia, intrauterine growth restriction and intrauterine fetal death were enrolled in post-natal wards.

### Exclusion criteria for couples were as follows

Smokers, couples with a history of thromboembolic events, chronic diseases such as diabetes mellitus, cardio-vascular diseases, inheritable and/or acquired thrombophilia, endocrine disorders, anatomic abnormalities of the uterus, endometriosis, hydrosalpinx and previous obstetric history with another partner (with a successful or a failed pregnancy) and any other significant medical history were excluded from the study. All participants were originally from Iran (north of Iran) with a shared common ethno-geographic and social origins.

### Thrombophilia panel

The male partners were screened for thrombophilia markers. Ten milliliters of venous blood was obtained, and the biochemical and genetic analyses were examined. In the biochemical panel, the following tests were performed: Activated protein C resistance (APCR), *antithrombin III (ATIII)*, Protein S and Protein C deficiency.

The ATIII activity in plasma was determined by a kinetic colorimetric method (Roche, 0525). Protein c activity was measured by a clotting method (*HYPHEN Biomed*, CKO31O, Hemoclot protein c) Likewise, the protein S activity was assessed by a clotting method (*HYPHEN Biomed*. Hemoclot Protein S, CKO41O). APCR was determined by a clotting assay (Stago, 00721). If the clotting time is shorter than 120 seconds, the APC-R test is considered positive.

For the thrombophilia genetic panel, all participants were requested to provide 10 mL whole peripheral blood, which was collected in ethylenediaminetetraacetic acid (EDTA) and stored at -20 ^°^C. DNA was extracted by QIAamp^®^ DNA Mini kit and followed by multiplex polymerase chain reaction (PCR) amplification. The genotyping was performed using a Devyser Thrombophilia kit (Art. No.: 8-A035) according to the manufacturer’s protocol, and the samples were run on an ABI3500 Genetic Analyzer. DNA polymorphism in Factor V Leiden G1691A, MTHFR C677T, and MTHFR A1298C was investigated. The summarized information on the polymorphic SNPs in this study has been demonstrated in Table 1.

**Table 1:** The summarized polymorphism information of the investigated SNPs in current study.

Gene name (rs)	Genomic position (hg38)	Position	Amino acid change
**Factor V Lieden** G1691A (rs6025)	chr1:169549811 g.169549811 C>T	NM_000130.5:c.1601G>A	p.Arg534Gln
**MTHFR C677T** (rs1801133)	chr1:11796321 g.11796321 G>A	NM_005957.5: c.66 5C>T	p.Ala222Gly
**MTHFR A1298C** (rs1801131)	chr1:11794419 g.11794419T>G	NM_005957.4: c.1286A>C	p.Glu429Ala

### Statistical analysis

Statistical analysis was performed using the Statistics Package for Social Sciences software (version 22, SPSS, Chicago, IL, USA). The results of statistical analysis were expressed as mean±SD and also frequency and percentage. The genotypes were tested for Hardy-Weinberg equilibrium (HWE) for both the patient and control group using the Chi square test.

The frequencies of genetic polymorphisms, as well as the frequencies of ATIII, PS, and PC deficiencies in RIF participants and controls, were compared with the chi-square or Fisher’s exact test. All tests were two-tailed, and the difference was considered significant if *p*<0.05. For each factor, odds ratio (OR) and 95% confidence interval were estimated separately to determine the strength of the association between the paternal thrombophilic factors and the risk of developing RIF.

## RESULTS

Forty-two males from couples who suffered from RIF with 42 controls were recruited in this study. The mean age of the RIF participants was 36.76±5.47 years compared to 36.17±5.54 years of controls. There were no significant differences between the case and control couples in terms of age, and body mass index (Table 2).

**Table 2:** Clinical data of the study and control groups.

Variables	Groups		*p*-value
	Case n=42	Control n=42	
**Male age (year)**	36.76±5.47	36.17±5.54	0.62
**Female age (year)**	32.83±5.65	31.12±6.73	0.21
**Male BMI**	25.83±3.84	26.93±4.59	0.22
**Female BMI**	27.63±3.79	25.52±3.30	0.008

Values are expressed as mean ± SD.

A description of outcome variables of the thrombophilic factors (biochemistry panel) is given in Table 3. In the case group, the prevalence of coagulation Factor V activity was 42.9%. In 18 of 42 participants, the APC-R value was below 120 seconds, and the APC resistance rate was 42.9% in the case group; when compared with the control group 16.7% (7/42), the difference was statistically significant (*p*=0.008) (OR=3.75; 95% CI, 1.38, 10.12). The prevalence of protein C and protein S deficiencies in RIF patients were 4.8% (2/42) and 2.4% (1/42), respectively, and 0% (0/42) in controls.

**Table 3:** Frequency of thrombophilia factors in the control and RIF patients.

Thrombophilia factors	Case n=42	Control n=42	*p*-value	Odds ratio (95%CI)
APC-R	18 (48.9%)	7 (16.7%)	**0.008**	**3.75 (1.38, 10.12)**
Protein C deficiency	2 (4.8%)	0	0.15	-
Protein S deficiency	1 (2.4%)	0	0.50	-
Antithrombin III deficiency	8 (19%)	1 (2.4%)	**0.01**	9.64 (1.46,43.19)
Combined thrombophilia	19 (45.2%)	6 (14.2%)	**0.001**	4.95 (1.75-13.81)

In the case group, the prevalence of ATIII deficiency was 19% (8/42), whereas a prevalence of 2.4% (1/42) was found for ATIII deficiency in the control group, the difference was statistically significant (*p*=0.01).

Combined thrombophilia (two or more thrombophilic factors) was significantly higher in the RIF group, 45.2% (19/42), when compared with the control group, 14.2% (6/42) (*p*=0.001). We observed that paternal thrombophilia conferred more than four times the odds of developing RIF in couples where the women had no apparent predisposition for this complication (OR=4.95; 95% CI, 1.75-13.86) (Table 3).

Furthermore, Table 4 illustrates the frequency of the thrombophilic genetic polymorphisms between the two groups, including the mutations in FVL G1691A, MTHFR A1298C and MTHFR C677T.

**Table 4:** Distribution of MTHFR C677T, MTHFR A1298C and FVL G1691A polymorphisms in men and their associations to RIF.

Thrombophilia factors	Case n=42	Control n=42	*p*-value	Odds ratio (95%CI)
**Factor V Leiden G1691A** GG GA AA Allele frequency G Allele frequency A	38 (90.4%) 2 (4.8%) 2 (4.8%) 78 (92.9%) 6 (7.1%)	39 (92.9%) 3 (7.1%) 0 81 (96.4%) 3 (3.6%)	- 0.68 0.29 - 0.31	1 0.68 (0.1-4.32) 5.12 (0.23-110.36) 1 2 (0.5-8.59)
**MTHFR C677T** CC CT TT Allele frequency C Allele frequency T	26 (61.9%) 14 (33.3%) 2 (4.8%) 66 (78.6%) 18 (21.4%)	27 (64.3%) 14 (33.3%) 1 (2.4%) 68 (81%) 16 (19%)	- 0.93 0.58 - 0.7	1 1.03 (0.41-2.59) 2.07 (0.17-24.31) 1 1.16 (0.54-2.46)
**MTHFR A1298C** AA AC CC Allele frequency A Allele frequency C	16 (38.1%) 18 (42.9%) 8 (19%) 50 (59.5%) 34 (40.5%)	11 (26.2%) 22 (52.4%) 9 (21.4%) 44 (52.4%) 40 (47.6%)	- 0.25 0.43 - 0.35	1 0.56 (0.2-1.51) 0.61 (0.18-2.07) 1 0.74 (0.4-1.37)

Except for FVL G1691A in case group, the other groups satisfied Hardy-Weinberg equilibrium (HWE) expectations (*p*>0.05).

The result indicated that just wild-type homozygote GG and heterozygote GA genotypes appeared in FVL G1691A controls, and none of the healthy controls showed mutant AA genotype. Statistical analysis by logistic regression model revealed no significant association between case and control genotype frequencies and RIF risk (*p*=0.69) (Table 3). Likewise, none of MTHFR C677T, and MTHFR A1298C were statistically significant between the case and control groups.

## DISCUSSION

This study revealed that at least one thrombophilic factor was found in 50% (21/42) of male partners of RIF patients. A comparison of the frequency of specific DNA polymorphisms between the RIF group and controls revealed no significant differences. However, combined thrombophilia was detected in a considerably higher proportion of cases (42%) than in controls (14.2%).

Inherited and acquired thrombophilias have been suggested as a potential risk factor for RIF. The incidence of thrombophilia in RIF patients has been reported to range from 4% to 62%. However, most studies have focused on RIF patients and not their male partners (Safdarian *et al.*, 2014; Shaulov *et al.*, 2020). Thrombophilic genetic polymorphisms have been shown to be risk factors for recurrent pregnancy loss (RPL) by reducing perfusion of the intervillous space and leading to placental failure (Ata & Urman, 2016). Similarly, interest in thrombophilia investigations among RIF patients is largely driven by the findings of studies on RPL. It has been proposed that RIF may be caused by similar damage to the decidual or chorionic vessels, or a reduction in trophoblast invasiveness, as increased coagulability could theoretically affect embryo implantation, possibly through vascular occlusion (Simcox *et al*., 2015). However, the effect of these polymorphisms on RIF is a controversial issue, and there are conflicting reports about this. On the other hand, the inconsistent results regarding the *beneficial effect of anticoagulant* therapy among couples with RIF who carry thrombophilic defect, highlight the necessity of assessment of this association (Nelson & Greer, 2008; Qublan *et al*., 2008; Lodigiani *et al*., 2011; Nichols *et al*., 2020).

Azem *et al*. (2004) revealed a high prevalence of thrombophilia in women with RIF compared to controls (44% *vs*. 18.2%). They stated that MTHFR, FVL, and prothrombin deficiency are possibly RIF risk factors (Azem *et al*., 2004). Furthermore, an association between IVF-embryo transfers failure, and an increased incidence of thrombophilia has been reported by Grandone *et al*. (2001).

Several reports have shown that the genetic polymorphisms of FVL and prothrombin genes may contribute to implantation failure or fetal loss after IVF. Most investigators have focused on DNA polymorphisms in FVL, MTHFR A1298C, and MTHFR C677T (Bauduer & Lacombe, 2005) and they detected a considerable association (Aflalo *et al*., 2004; Azem *et al*., 2004; Coulam *et al*., 2006a;b). However, other studies have not been able to confirm this association (Kutteh *et al*., 1999; Steinvil *et al*., 2012). It should be noted that most of these studies had a small sample size, only focused on female carriers and included information that is *clinically irrelevant* such as MTHFR heterozygous mutation.

Some studies have demonstrated a higher risk of infertility (Behjati *et al*., 2006) and implantation failure (Grandone *et al*., 2001) among FVL carriers. However, other studies have found that FVL-carrying males (rs6025) have 3.5-fold more fecundity than non-carriers (Van Mens *et al*., 2017), and that the overall rate of successful embryo transfers was higher in FVL carriers (Göpel *et al*., 2001), but the mechanism behind the association remains elusive. It is worth mentioning that the prevalence of the FVL mutation varies by ethnicity, with the highest rates being seen in European populations (2%-8%) (Raptopoulou *et al*., 2022). The highest frequency of the FVL G1691A mutation was reported in Mediterranean countries, with a prevalence of up to 15% in some populations (Bauduer & Lacombe, 2005).

On the basis of current study, we observed a deviation from Hardy-Weinberg equilibrium for FVL G1691A in case group; which can regard as a signal of true association. However, bigger sample size is needed to determine this causation (Table 4).

Few studies have reported allele frequencies (2.97, 4.1 and 6.4) in Iranian populations, however, in our study, it was 7.1% in the RIF group, and 3.6% in controls (Table 3), and we did not find a considerable difference that supports evidence from previous observations among Iranian populations (Rahimi *et al*., 2008; Karimi *et al*., 2009; Houjaghani & Ghorbani, 2022).

Successful *implantation requires* the coordination *between* a *healthy embryo* and a functionally competent and receptive endometrium. Failure of implantation due to embryonic causes is associated with either genetic abnormalities or inherited thrombophilia that impair the embryo to develop in utero, and implant (Rogenhofer *et al*., 2021). As half of the fetal genes are inherited from the father, it has been hypothesized that paternal thrombophilic alleles play a role in adverse assisted reproductive technology (ART) outcomes (Simon & Laufer, 2012).

Udry *et al*. (2014) showed that paternal FV Leiden carriage conferred more than six fold the risk of developing RPL in couples where the women had no apparent predisposition for this obstetric complication. In this study we did not find a considerable difference in the frequency of paternal FVL G1691A mutation among RIF and control groups.

Coulam *et al*. (2006b) revealed that multiple thrombophilic gene mutations rather than the specific gene in either partner of RPL couples increased 1.9-fold the risk of miscarriages in subsequent pregnancies. They also assessed the prevalence of 10 thrombophilic gene mutations in RIF patients and concluded that the total number of thrombophilic gene mutations is higher among RIF patients than controls (Coulam *et al*., 2006a). This finding confirms the concept that some coagulation factors may have non-hemostatic roles during implantation. One argument is that genetic studies are influenced by the ethnic group, and the low prevalence of some thrombophilic gene mutations in the Iranian population makes it difficult to identify the paternal contribution to recurrent implantation failure (RIF). The minor allele frequency of the FVL G1691A mutation (rs6025) is 0.015 in the Iranian population (www.iranome.ir).

MTHFR is an essential enzyme in the folate metabolism pathway, and is involved in *DNA synthesis and methylation.* The A1298C and C677T are the most common single nucleotide polymorphisms (SNPs) in the MTHFR gene (24). A meta-analysis by Yang et al., found that the paternal C677T and A1298C MTHFR polymorphisms were associated with an increased risk of RPL; They also found a considerable correlation between fetal MTHFR A1298C polymorphism and RPL but not C677T. Choi *et al*. (2016) reported that the MTHFR A1298C and C677T genotypes may be associated with an increased risk of RIF. However, their study only investigated the role of these genotypes in women with maternal thrombophilia.

Despite these results, Toth *et al*. (2008) observed the incidence of the paternal mutations in the FVL G1691 A, and MTHFR C677T were higher in the control group, and concluded that paternal thrombophilia is not associated with early miscarriage. Our study also found no considerable difference in the MTHFR gene mutations between the two groups. It is important to note that the association of paternal thrombophilia with pregnancy complications is likely to be lower, as paternal thrombophilia can only have an adverse effect if the fetus inherits the thrombophilic allele. This means that the fetus must have the same mutation as the father in order to be affected.

In our study, the incidence of APCR was 48.9% which was meaningfully more prevalent than the controls (16.7%). The incidence of APCR in this study was also higher than the previously reported incidence in Iranian couples with recurrent abortion (21.3%) (Matin *et al*., 2019). Some investigators have shown that APCR is more common in men than in women. Takhviji *et al.* (2021) found that APCR is 2.3 times more likely in men. Therefore, it is important to investigate thrombophilic factors in both partners of couples with RIF. While our study observed a relatively low prevalence of protein C and protein S deficiencies in both the RIF and control groups, it is important to acknowledge that these deficiencies are generally more prevalent in patients with certain risk factors and that their prevalence varies among different ethnic groups.

Although we observed that the overall frequency of APCR and ATIII deficiencies and combined thrombophilia were higher in the RIF group than in the controls. However, we cannot be sure whether the failure of implantation in these couples was caused by paternal thrombophilia, as fetal thrombophilia may also affect IVF/ICSI-embryo implantation failure.

The findings of this study may be limited by the small sample size, which may not be large enough to detect significant differences between the case and control groups. Additionally, the study identified three specific single-nucleotide polymorphism (SNP) polymorphisms in the FVL and MTHFR genes. However, mutations in other regions of these genes may also impact the biological functions of the proteins they encode. Therefore, it is recommended to prenatally identify mutations in other regions of the FVL and MTHFR genes. Moreover, the study was a case-control study. case-control studies are more susceptible to bias than other types of studies.

Despite aforementioned limitations, this study has some strength: (1) to the best of our knowledge, this is the first to elucidate the association of paternal thrombophilia with RIF risk. (2) The cases in this study consisted of a strictly selected group of homogenous couples from the Iranian population with no probable cause of this obstetric problem (3) Most of the studies have only focused on the genetic thrombophilia panel of women with RIF, however in this study both *genetic* and biochemical panel of parental *thrombophilia* were evaluated.

## CONCLUSION

The findings of this study suggest that paternal thrombophilia may be a risk factor for recurrent implantation failure (RIF). This is important because it could help identify couples at higher risk for RIF and guide their management. In future studies, it is important to address the limitations of the current study. Specifically, the findings of this study should be validated in larger studies with more diverse populations. Additionally, future studies should investigate the mechanisms by which paternal thrombophilia leads to RIF. This could lead to the development of new targeted treatments for couples with paternal thrombophilia. Future research should investigate not only thrombophilic genetic polymorphisms, but also biochemical biomarkers in both couples with RIF. However, given the small sample size of the current study, caution must be applied and further studies are needed to support these assumptions

## References

[r1] Aflalo ED, Sod-Moriah UA, Potashnik G, Har-Vardi I. (2004). Differences in the implantation rates of rat embryos developed in
vivo and in vitro: possible role for plasminogen activators. Fertil Steril.

[r2] Ata B, Urman B. (2016). Thrombophilia and assisted reproduction technology-any
detrimental impact or unnecessary overuse?. J Assist Reprod Genet.

[r3] Azem F, Many A, Ben Ami I, Yovel I, Amit A, Lessing JB, Kupferminc MJ. (2004). Increased rates of thrombophilia in women with repeated IVF
failures. Hum Reprod.

[r4] Bashiri A, Halper KI, Orvieto R. (2018). Recurrent Implantation Failure-update overview on etiology,
diagnosis, treatment and future directions. Reprod Biol Endocrinol.

[r5] Bauduer F, Lacombe D. (2005). Factor V Leiden, prothrombin 20210A, methylenetetrahydrofolate
reductase 677T, and population genetics. Mol Genet Metab.

[r6] Behjati R, Modarressi MH, Jeddi-Tehrani M, Dokoohaki P, Ghasemi J, Zarnani AH, Aarabi M, Memariani T, Ghaffari M, Akhondi MA. (2006). Thrombophilic mutations in Iranian patients with infertility and
recurrent spontaneous abortion. Ann Hematol.

[r7] Choi Y, Kim JO, Shim SH, Lee Y, Kim JH, Jeon YJ, Ko JJ, Lee WS, Kim NK. (2016). Genetic Variation of Methylenetetrahydrofolate Reductase (MTHFR)
and Thymidylate Synthase (TS) Genes Is Associated with Idiopathic Recurrent
Implantation Failure. PLoS One.

[r8] Coulam CB, Jeyendran RS, Fishel LA, Roussev R. (2006a). Multiple thrombophilic gene mutations are risk factors for
implantation failure. Reprod Biomed Online.

[r9] Coulam CB, Jeyendran RS, Fishel LA, Roussev R. (2006b). Multiple thrombophilic gene mutations rather than specific gene
mutations are risk factors for recurrent miscarriage. Am J Reprod Immunol.

[r10] Di Nisio M, Rutjes AW, Ferrante N, Tiboni GM, Cuccurullo F, Porreca E. (2011). Thrombophilia and outcomes of assisted reproduction technologies:
a systematic review and meta-analysis. Blood.

[r11] Fishel S, Patel R, Lytollis A, Robinson J, Smedley M, Smith P, Cameron C, Thornton S, Dowell K, Atkinson G, Shaker A, Lowe P, Kazem R, Brett S, Fox A. (2014). Multicentre study of the clinical relevance of screening IVF
patients for carrier status of the annexin A5 M2 haplotype. Reprod Biomed Online.

[r12] Göpel W, Ludwig M, Junge AK, Kohlmann T, Diedrich K, Möller J. (2001). Selection pressure for the factor-V-Leiden mutation and embryo
implantation. Lancet.

[r13] Grandone E, Colaizzo D, Lo Bue A, Checola MG, Cittadini E, Margaglione M. (2001). Inherited thrombophilia and in vitro fertilization implantation
failure. Fertil Steril.

[r14] Houjaghani S, Ghorbani A. (2022). The association of factor V Leiden mutation (G1691A) with
pregnancy complications (miscarriage) in the Iran, East
Azerbaijan. Clin Exp Health Sci.

[r15] Karimi M, Panahandeh Shahraki GR, Yavarian M, Afrasiabi A, Dehbozorgian J, Bordbar M, Mannucci PM. (2009). Frequency of factor V Leiden and prothrombin polymorphism in
south of Iran. Iran J Med Sci.

[r16] Kutteh WH, Park VM, Deitcher SR. (1999). Hypercoagulable state mutation analysis in white patients with
early first-trimester recurrent pregnancy loss. Fertil Steril.

[r17] Lodigiani C, Di Micco P, Ferrazzi P, Librè L, Arfuso V, Polatti F, Benigna M, Rossini R, Morenghi E, Rota L, Brenner B, Setti PE. (2011). Low-molecular-weight heparin in women with repeated implantation
failure. Womens Health.

[r18] Manna C, Lacconi V, Rizzo G, De Lorenzo A, Massimiani M. (2022). Placental Dysfunction in Assisted Reproductive Pregnancies:
Perinatal, Neonatal and Adult Life Outcomes. Int J Mol Sci.

[r19] Matin M, Bashash D, Hasrak K, Baghestani A, Hamidpour M. (2019). Frequency of protein C, S and antithrombin III deficiency and
presence of antibodies of antiphospholipid syndrome in women with recurrent
abortion. Iran J Obstet Gynecol Infertil.

[r20] Nelson SM, Greer IA. (2008). The potential role of heparin in assisted
conception. Hum Reprod Update.

[r21] Nichols KM, Henkin S, Creager MA. (2020). Venous Thromboembolism Associated With Pregnancy: JACC Focus
Seminar. J Am Coll Cardiol.

[r22] Qublan H, Amarin Z, Dabbas M, Farraj AE, Beni-Merei Z, Al-Akash H, Bdoor AN, Nawasreh M, Malkawi S, Diab F, Al-Ahmad N, Balawneh M, Abu-Salim A. (2008). Low-molecular-weight heparin in the treatment of recurrent IVF-ET
failure and thrombophilia: a prospective randomized placebo-controlled
trial. Hum Fertil (Camb).

[r23] Rahimi Z, Vaisi-Raygani A, Mozafari H, Kharrazi H, Rezaei M, Nagel RL. (2008). Prevalence of factor V Leiden (G1691A) and prothrombin (G20210A)
among Kurdish population from Western Iran. J Thromb Thrombolysis.

[r24] Raptopoulou A, Michou V, Mourtzi N, Papageorgiou EG, Voyiatzaki C, Tsilivakos V, Beloukas A, Bei TA. (2022). Large-scale screening for factor V Leiden (G1691A), prothrombin
(G20210A), and MTHFR (C677T) mutations in Greek population. Health Sci Rep.

[r25] Rogenhofer N, Markoff A, Ennerst X, Bogdanova N, Thaler C. (2021). Maternal and paternal carriage of the annexin A5 M2 haplotype: a
possible risk factor for recurrent implantation failure
(RIF). J Assist Reprod Genet.

[r26] Safdarian L, Najmi Z, Aleyasin A, Aghahosseini M, Rashidi M, Asadollah S. (2014). Recurrent IVF failure and hereditary
thrombophilia. Iran J Reprod Med.

[r27] Shaulov T, Sierra S, Sylvestre C. (2020). Recurrent implantation failure in IVF: A Canadian Fertility and
Andrology Society Clinical Practice Guideline. Reprod Biomed Online.

[r28] Simcox LE, Ormesher L, Tower C, Greer IA. (2015). Thrombophilia and Pregnancy Complications. Int J Mol Sci.

[r29] Simon A, Laufer N. (2012). Repeated implantation failure: clinical approach. Fertil Steril.

[r30] Somigliana E, Vigano P, Busnelli A, Paffoni A, Vegetti W, Vercellini P. (2018). Repeated implantation failure at the crossroad between
statistics, clinics and over-diagnosis. Reprod Biomed Online.

[r31] Steinvil A, Raz R, Berliner S, Steinberg DM, Zeltser D, Levran D, Shimron O, Sella T, Chodick G, Shalev V, Salomon O. (2012). Association of common thrombophilias and antiphospholipid
antibodies with success rate of in vitro fertilisation. Thromb Haemost.

[r32] Takhviji V, Zibara K, Maleki A, Azizi E, Hommayoun S, Tabatabaei M, Ahmadi SE, Soleymani M, Ghalesardi OK, Farokhian M, Davari A, Paridar P, Kalantari A, Khosravi A. (2021). A case-control study on factor V Leiden: an independent,
gender-dependent risk factor for venous thromboembolism. Thromb J.

[r33] Tanacan A, Beksac MS. (2019). Spontaneous pregnancies in patients with at least one failed IVF
cycle after the management of autoimmune disorders, hereditary
thrombophilia, and methylation disorders. JBRA Assist Reprod.

[r34] Toth B, Vocke F, Rogenhofer N, Friese K, Thaler CJ, Lohse P. (2008). Paternal thrombophilic gene mutations are not associated with
recurrent miscarriage. Am J Reprod Immunol.

[r35] Udry S, Aranda FM, Latino JO, de Larrañaga GF. (2014). Paternal factor V Leiden and recurrent pregnancy loss: a new
concept behind fetal genetics?. J Thromb Haemost.

[r36] van Dunné FM, de Craen AJ, Heijmans BT, Helmerhorst FM, Westendorp RG. (2006). Gender-specific association of the factor V Leiden mutation with
fertility and fecundity in a historic cohort. The Leiden 85-Plus
Study. Hum Reprod.

[r37] van Mens TE, Joensen UN, Bochdanovits Z, Takizawa A, Peter J, Jørgensen N, Szecsi PB, Meijers JCM, Weiler H, Rajpert-De Meyts E, Repping S, Middeldorp S. (2017). Factor V Leiden is associated with increased sperm
count. Hum Reprod.

[r38] Yapijakis C, Pachis N, Avgoustidis D, Adamopoulou M, Serefoglou Z. (2016). Mutations of Two Major Coagulation Factors Are Not Associated
with Male Infertility. In Vivo.

[r39] Younis JS, Ben-Ami M, Izhaki I, Brenner B, Sarig G. (2014). Reduced protein C Global assay level in infertile women prior to
IVF-ET treatment. J Assist Reprod Genet.

